# Critical Minerals for Zero-Emission Transportation

**DOI:** 10.3390/ma15165539

**Published:** 2022-08-12

**Authors:** Frank Czerwinski

**Affiliations:** CanmetMATERIALS, Natural Resources Canada, Hamilton, ON L8P 0A5, Canada; frank.czerwinski@nrcan-rncan.gc.ca

**Keywords:** critical minerals, zero-emission vehicles, electric vehicles, clean energy, circular economy, recycling

## Abstract

Fundamentals of critical minerals and their paramount role in the successful deployment of clean energy technologies in future transportation are assessed along with current global efforts to satisfy the needs of automotive supply chains and environmental concerns. An implementation of large quantities of minerals, in particular metals, into the manufacturing of strategic components of zero-emission vehicles will bring new challenges to energy security. As a result, a reduced dependency on conventional hydrocarbon resources may lead to new and unexpected interdependencies, including dependencies on raw materials. It is concluded that to minimize the impact of a metal-intensive transition to clean transportation, in addition to overcoming challenges with minerals mining and processing, further progress in understanding the properties of critical materials will be required to better correlate them with intended applications, to identify potential substitutions and to optimize their use through the sustainable exploration of their resources and a circular economy.

## 1. Introduction

The zero-emission transportation is a paramount part of the anticipated transition to a more sustainable, low-carbon economy, powered by clean energy technologies that differ profoundly from solutions relying on fossil fuels. The inherent feature of a new energy strategy is a substantially higher, often multiple times, demand for minerals than that seen in their conventional counterparts. It is frequently quoted from the International Energy Agency (IEA) that the typical electric car requires six times the mineral inputs of a conventional car and an onshore wind plant requires nine times more mineral resources than a gas-fired plant [1]. In another powerful comparison, the photovoltaic energy is portrayed as requiring up to 40 times more copper than the fossil-fuel combustion [2]. According to the IEA prediction, to reach the climate stabilization at a well below 2 °C global temperature rise by 2050, it would require six times more mineral inputs for clean energy technologies in 2040 than today. Thus, while previously the energy sector represented only a small portion of the total minerals demand, it is emerging as the major force in mineral markets and as a result of this transformation, minerals bring new challenges to the energy security. Although the metal-intensive transition to the net-zero economy will reduce dependencies on conventional hydrocarbon resources, it may lead to new and unanticipated interdependencies, including dependencies on raw materials [3]. 

Due to the anticipated exponential growth of zero-emission transportation, large quantities of minerals, in particular metals, will be required for vehicle manufacturing. Many of them, including lithium, graphite, rare earth elements, cobalt, nickel, copper or platinum group metals, are generally classified as the critical minerals. They have a significant economic importance and their insecure supplies may hinder the development and implementation of new technologies. Thus, the successful deployment of clean technologies in future transportation will require further progress in understanding the materials properties to better correlate them with applications, enabling potential substitutions and optimizing their use through a sustainable exploration of their resources and a circular economy. This report provides the fundamentals of critical minerals, their role in zero-emission transportation and assesses the current global challenges with satisfying the needs of automotive supply chains and environmental concerns. 

## 2. Critical Minerals

A fundamental principle of sustainable economic development and smooth manufacturing cycle, particularly in well-established economies, is the uninterrupted supply chain with raw materials that is free from disturbances and bottlenecks, contributing to the pricing instability and market volatility, thus disrupting the production [4]. This condition is valid for a variety of technologies since raw materials play an important role in defense, the economy, renewable energy development and infrastructure. Growing reliance on raw materials has intensified the competition to identify new resources and establish stable, long-term supply chains. Although the increasing interest in minerals resources, caused by global competition, reached such enormous attention in recent years, the subject is not new and has been considered for decades [5]. 

The minerals awareness was initiated after World War I as a warranty of sustaining military power. The term “strategic and critical minerals” was used as early as 1938 when emphasizing that the industrial nations should be self-sufficient in regard to certain materials, portrayed as the “materials essential in the promotion of modern warfare” [6]. Similar terminology was used in the “Strategic and Critical Materials Stock Piling Act” of 1939 [7], when political conflicts dominated concerns about the security of raw materials supplies. At present, in the USA and Canada the term “critical minerals” is used while “critical raw materials” (CRM) is used by the European Union. The other terminology includes “strategic minerals” or “advantageous minerals” used by China. 

### 2.1. Defining Minerals Criticality

Minerals criticality is a subjective concept that has been shaped throughout decades. A number of criticality studies were conducted in an effort to define a consensus of currently critical materials, essentially defining the modern criticality paradigm, which allows the interpretation of local perspectives in a global context [8]. The supplies of critical minerals are recognized to be a subject of great societal and environmental risk and uncertainty. It should be emphasized that the scarcity of a mineral in the Earth’s crust alone does not automatically make it critical. A classification of the mineral as critical is derived through its importance to key industry sectors or applications and the overall functioning of the country economy. The criticality criteria may vary across geographical regions, though the essence remains similar. According to [9], material criticality is defined as “economic and technical dependency on a certain material, as well as the probability of supply disruptions, for a defined stakeholder group within a certain time frame”. According to the IEA, the classification of materials as critical is based on [1]:

- Their significant economic importance for key sectors in the regional economy and/or national security;
- A high-supply risk due to the very high import dependence and high level of concentration of a set of critical raw materials in particular countries;
- A lack of viable substitutes, due to the very unique and reliable properties of these materials for existing, as well as future applications.

Due to growing concerns about the supply security of raw materials, the concept termed as “raw material criticality” is at the center of the attention. Over the years since the concept of criticality was formalized, a number of evaluation methods of critical material risks were tested. The potential supply risks come from different sources: depletion due to mineral scarcity, geographical concentration of deposits, political stability of producing countries, geopolitical risks in global raw materials trade as well as low recycling rates. 

### 2.2. Geopolitical Factors and National Lists

**Figure 1:**
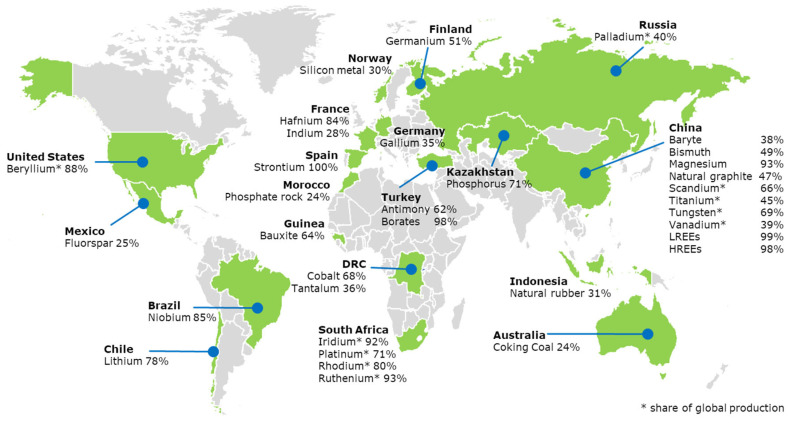
Global location of critical minerals with specified major supplier countries to the European Union, e.g., China provides 98% of rare earth elements (REE), Turkey provides 98% of borates and South Africa provides 71% of the platinum (2020) [11].

The geographical concentration of minerals creates a major strategic issue: a high global demand versus limited local supply. Since minerals originate from a limited number of mining sites and nations, they are highly vulnerable to supply restrictions [10]. In this respect, the supply risk is always a key factor to determine the criticality in countries that have a high dependency on imported minerals. As a result, there is a potential insecurity in how markets are responding and what roles governments should play to secure the supply chains. An example of critical minerals supply for the European Union is shown in Figure 1.

Minerals criticality is commonly evaluated from a regional and geopolitical perspective. In practice, the extraction, refining and use of various materials used in modern transportation vehicles involve complex interactions through associated mining and global trade companies, governments and industrial chains, flowing up to supply chains of then manufactured components for use by vehicle manufacturers. Therefore, the security of supply issues of some minerals is seen through a geographical and political perspective rather than reflecting the actual lack of supply of the mineral in question [12]. Based on resources available, countries can be divided into resource-poor and resource-rich. The resource-poor countries recognized critical mineral supply as a strategic issue, made investments designed to diversify supplies, develop substitutes, improve reuse and recycling and covered them by government policies. In practice, no country is fully self-sufficient in meeting all its mineral resource needs. There are countries, however, where some of the strategic minerals are not subject to a shortage of supply, at least until the near future. In this case, the exploration of critical materials is protected and secured as “strategic” to hold a domestic resource advantage. Having an abundance of critical minerals, a country will act as a powerhouse in supplying the global market. Thus, individual countries have their own lists of critical minerals. It is of interest that as seen in Table 1 and discussed below, the lists of critical minerals specified by individual countries are quite similar with common overlaps.

**Canada:** The Critical Minerals List, unveiled on 11 March 2021 and containing 31 minerals, all of which are available in Canada, is considered integral to the Canadian economy [13]. The list highlights focus areas for future Canadian mining policies and investments and builds on the existing Canadian Minerals and Metals Plan. It was developed by Natural Resources Canada, using a criteria-based approach and in consultation with provinces, territories as well as exploration, mining and manufacturing industries and associations. 

**USA:** The Energy Act of 2020 defines a “critical mineral” as a nonfuel mineral or mineral material essential to the economic or national security of the U.S. and which has a supply chain vulnerable to disruption. The 2022 list of critical minerals, released by The United States Geological Survey after an extensive multiagency assessment contains 50 mineral commodities critical to the U.S. economy and national security [14]. The list contains 15 more commodities compared to the nation’s first list of critical minerals created in 2018, mainly due to splitting the rare earth elements and platinum group elements into individual entries rather than including them as “mineral groups.” In addition, the 2022 list adds nickel and zinc while removing helium, potash, rhenium and strontium.

**European Union:** The European Commission carried out a criticality assessment at EU level on a wide range of nonenergy and nonagricultural raw materials based on a methodology for establishing the list of critical raw materials [15]. The EU list that contained 14 materials in 2011, was revised to 20 materials in 2014, then to 27 in 2017 and to 30 in 2020. The 2020 criticality assessment was carried out for 66 candidate materials (63 individual materials and 3 material groups: heavy rare earth elements, light rare earth elements, platinum group metals), amounting to 83 materials in total [11]. In addition, the European Commission included borate, coking coal, natural rubber, phosphate rock, phosphorus and silicon metal into the 2020 Critical Raw Materials list.

**China:** The difference in criticality assessments in China is caused by the consideration of the factor of supply risk. China’s dominance in the critical minerals space suggests that some raw materials are considered “strategic” for reasons unrelated to supply risk because China has them in abundance. China’s first official policy and catalogue of “strategic minerals” was established in 2016 by the Ministry of Land and Resources [16]. The metallic minerals list was refined in 2018 to include four subgroups: (1) the noble metals of Li, Be, Rb, Cs, Nb, Ta, Zr, Hf and W; (2) the rare earth metals of La, Ce, Pr, Nd, Sm, Eu, Gd, Tb, Dy, Ho, Er, Tm, Yb, Lu, Sc and Y; (3) the companion metals of Ga, Ge, Se, Cd, In, Te, Re and Tl; and (4) the precious metals of PGMs, Cr and Co [17,18].

**Japan:** As a resource-scarce country, Japan has widely been considered as particularly vulnerable to resource restriction since it is heavily reliant on imported raw materials and needs a strategy to secure its supply chain [19]. Japan’s 2018 list comprises 34 critical minerals, 4 up from 30 in 2012. Since 2016, the Ministry of Economy, Trade and Industry (METI) has started criticality assessments of metals used in the Japanese industry by applying the approaches employed in the US and the EU, preparing guidelines to support criticality assessments conducted by companies. The supply-chain management of critical minerals at the corporate level is seen increasingly important, and the guidelines are designed to promote more companies to evaluate the raw materials criticality.

**Australia:** The focus of the Critical Minerals Association of Australia has historically been on minerals that are deemed critical by other sovereign entities, and on Australia’s potential to contribute to the global supply chain of these critical minerals. Given that Australia is known to export much greater quantities of minerals than it imports, this focus on an increased export potential seems obvious. The 2022 updated strategy builds on the first Critical Minerals Strategy, published in 2019 [20]. It has a vision to put Australia at the center of meeting the growing demand for critical minerals. Australia’s current list of 26 critical minerals highlights its priority critical minerals and is based on global technology needs, particularly around electrification, advanced manufacturing and defense.

### 2.3. Assessment of Critical Materials Supply Risks

**Figure 2:**
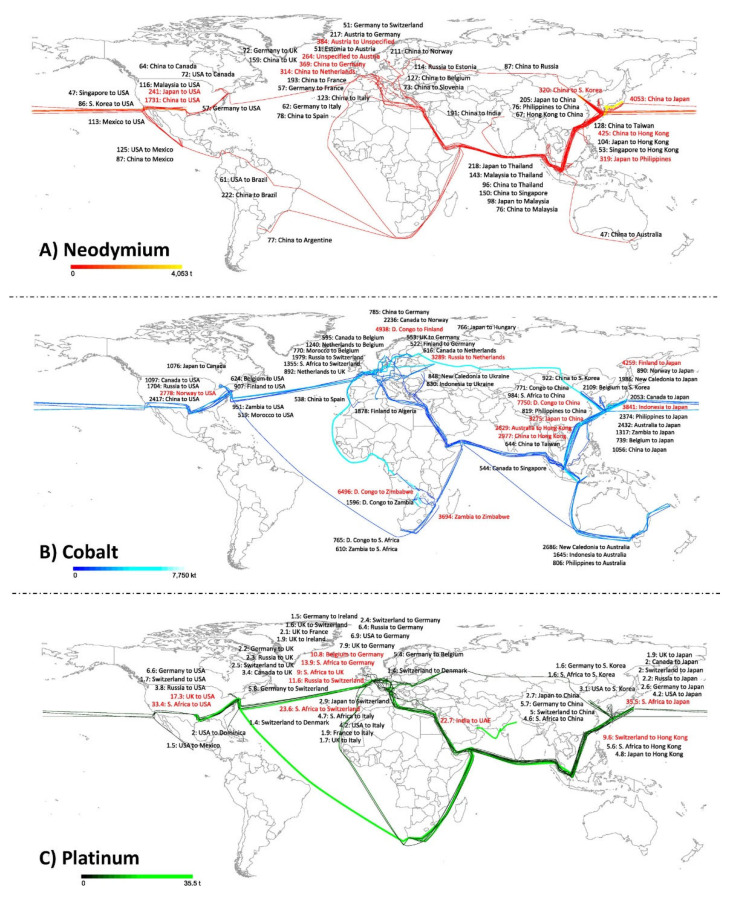
Global flow of critical minerals neodymium (**A**), cobalt (**B**) and platinum (**C**) through international trade in 2005. For the top 10 flows, the countries and volumes of mineral involved (tonne for neodymium and platinum, 10^3^ × tonne for cobalt are indicated in red [21].

Due to considerable concern about supply risks, it is important to determine how individual countries in the global economy are involved in the flows of critical minerals and determine their strategic responsibilities. An example in Figure 2 shows the complexity of international trade and global flows of 18.6 kt of neodymium, 154 kt of cobalt and 402 t of platinum. It also identifies the main commodities with the top 50 bilateral trade links associated with these metals [21].

A capability of quantifying the implications of prospective manufacturing trends for raw material resources is valuable in order to control the efficient resource management. At present, there are specific methodologies used for assessment of the product-level supply risk [22] with examples described in [23]. Although the general issues of supply security are commonly accepted, there is no unified approach to their assessment methods with some authors pointing to a lack of adherence to risk theory [24]. The mineral supply and related risks are highly dynamic and constantly evolving due to geopolitics and trade tensions, metallurgical and extractive advances and technological innovations. Therefore, mineral markets are continuously analyzed, and new methods are developed to determine the supply chain resilience and to predict potential issues well in advance.

**Figure 3:**
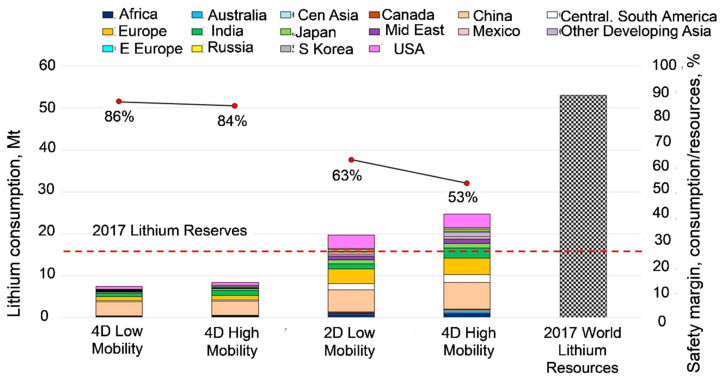
Modeling the critical minerals consumption using the TIMES Integrated Assessment Model (TIAM-IFPEN version): comparing the cumulated lithium consumption (2005–2050) and the world resources in 2017. As modeled, only 2 °C scenarios will reach cumulative lithium consumption higher than the 2017 level of lithium reserves (red dotted line); for a 4 °C scenario, the cumulated lithium demand is far under the 2017 reserves [25].

As an example, the TIMES Integrated Assessment Model (TIAM-IFPEN version) was used to assess the lithium supply for the production of EVs [25]. Through incorporating an endogenous representation of the lithium supply chain, its dynamic criticality was determined based on several paths of climate and/or mobility scenarios. The modeling outcome in Figure 3 shows that the cumulated demand over the period 2005–2050 reaches up to 53% of the current resources in the 2 °C scenario with a high mobility of 75% of the worldwide fleet. Although there was an absence of geological criticality, certain forms of metal supply vulnerability were revealed. The issue is that world lithium resources are estimated at 22 Mt [26] so for both scenarios, the predicted Li consumption would exceed it.

## 3. Material Criticality and Circular Economy

A circular economy considers the full life cycle of materials and offers opportunities for more a productive use of materials through recirculating their larger share through reuse and recycling, reducing waste in production, extending the lifetime of products and through associated policies. This contrasts with the linear economy and its predisposition towards wasting valuable resources. The circular economy has a particular meaning for critical minerals; in contrast to an emphasis on recycling at the end of life for conventional materials, for critical minerals the waste reduction is controlled throughout the entire cycle starting from extraction, where waste is subjected to further processing to extract lesser-concentrated critical minerals. Recycling is an important source of secondary raw materials and reduces the dependency on primary raw materials.

### 3.1. Impact of Critical Minerals Extraction and Processing

Mineral mining and processing facilities may create environmental challenges. Mining involves the extraction of rocks containing minerals, transport to processing plants, separating and refining the metals and then disposing of the waste. The mining infrastructure consumes large amounts of energy and water, generating air pollution and hazardous waste. Managing the downside risks that accompany minerals extraction sits at the core of a just transition. To prevent conflicts, a detailed analysis of the mining cycle is required, from an initial survey and exploration of mineral deposits to the mine closure. Modern mining operations control and mitigate the potential environmental consequences of extracting metals, and such operations should strictly be regulated [27]. The key to effective mitigation lies in implementing the scientific and technological advances that prevent the undesired environmental impacts. Operations and waste products associated with metal extraction and processing are the principal causes of environmental concerns, that typically include [28]:

- Physical disturbances to the landscape;
- Soil and water contamination;
- Air contamination;
- Public safety.

**Figure 4:**
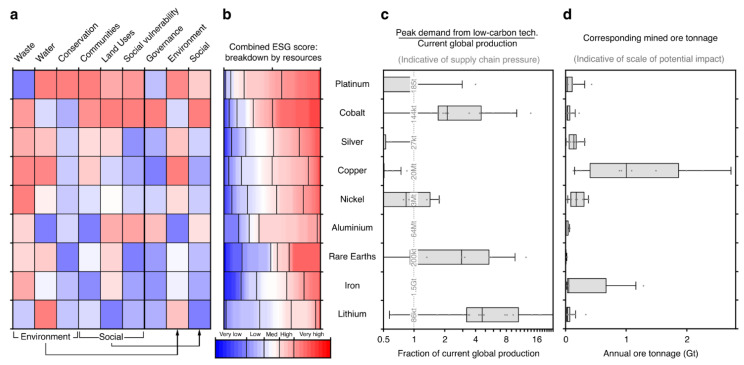
Risk profiles and demand projections for energy transition metals: (**a**) environmental, social and governance risk matrix ranked by total score, defined as the sum of the scores for the seven dimensions (first seven columns); (**b**) breakdown of total risk scores by resource tonnage where colors correspond to risk levels; (**c**) projected demand associated with growth in low-carbon energy technologies for considered metals, as a fraction of current global production; (**d**) approximate mined ore tonnage corresponding to specified demand [29].

Past mismanagements of environmental, social and governance risks have created social and environmental pressures within mineral-resource-rich regions. According to [29], the future metal production faces a dual pressure: an increased demand to support the clean energy transition and an increased scrutiny due to adverse impacts in locations with pre-existing environmental, social and governance complexity. An example of a detailed, multidirectional risk assessment involving a variety of factors is shown in Figure 4.

New projects in sound governance jurisdictions will have to confirm their ability to assess, manage and minimize environmental, social and governance risks, or face opposition, which may, in turn, constrain the supply of energy transition metals and inhibit the clean energy transition [30]. Historically, mining has faced the environmental impacts, but new approaches could help reduce them as the world needs more metals for renewable energy.

### 3.2. Circularity as a Solution for Criticality

**Figure 5:**
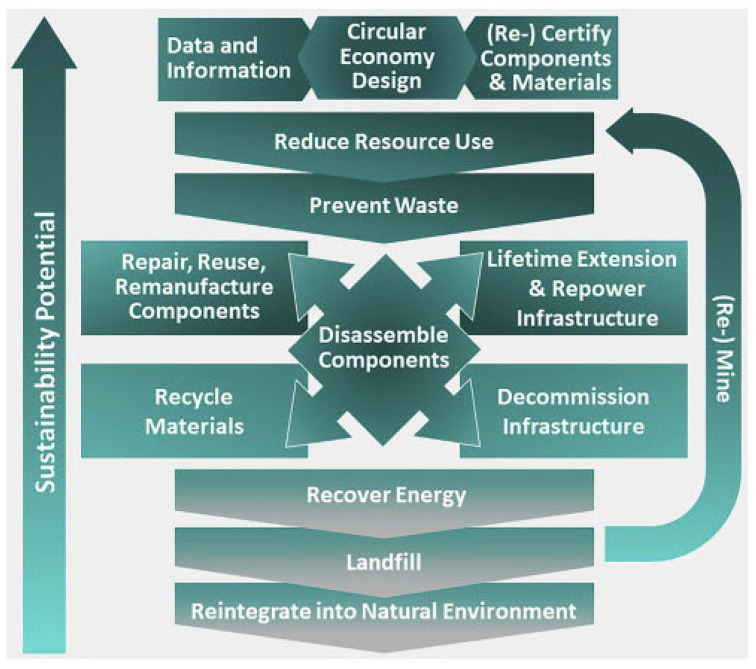
Circular economy strategies to reduce the criticality of materials applied in clean energy transition [31].

The unique sustainability challenges, presented by the growing need for critical minerals, require new approaches to minimize the production and consumption impacts. As portrayed in Figure 5, the circular economy offers a variety of solutions that may help minimize the criticality risks and improve the sustainability of critical minerals extraction, use and recycling, preventing wastes, slowing resource flows, re-storing resources in landfills that can be converted into valuable minerals [31].

**Figure 6:**
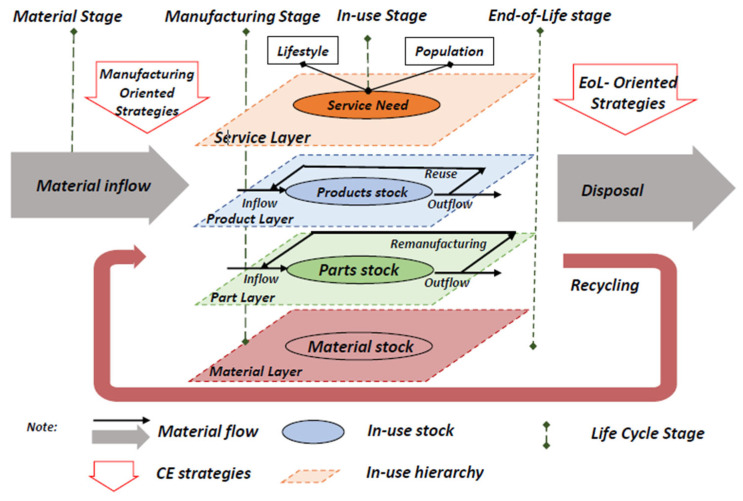
The analytical framework for integrated circular economy of critical material management. The framework includes four life-cycle stages from material to end-of-life stage, and in-use stage is specified with four hierarchical layers in which different strategies can be applied. The implementation of two critical sets of circular economy, manufacturing and end-of-life stage, is highlighted [32].

Building a circular economy for critical materials involves the necessity of manufacturing-oriented strategies. The new paradigm for addressing the metal criticality emphasizes the role of design that enables material and product reuse, upgrade, remanufacturing and recycling. The essence of this paradigm is that the circularity starts from product design, with the use of secondary materials in overall value chains [32]. The strategies of a circular economy include the evaluations of material requirements at the R&D stage, sustainable material selection, design of resilient supply chains, design for disassembly, durability and recycling, circular business models, material tracking technology and circularity passports [33]. The concept that enables the circular economy solutions and minimizes the risks of material criticality is implemented through three interconnected approaches of narrowing, slowing and closing resource loops [33]:

- Narrowing resource loops—this refers to resource-efficient processes being able to fulfill societal needs but at the same time reducing the net quantity of materials used per unit of economic activity;
- Slowing resource loops—this refers to methods used to retain the use and value of a material or product for as long as possible; it includes designing products that are durable and retain both their function and their appeal to users over an extended lifespan;
- Closing resource loops—this refers to the processes used to recover a resource at the end of their lifespan, and send it back to productive use, as shown in Figure 6.

All the assessments show that the circular economy is critical for the sustainable deployment of clean transportation technologies. Recycling is also an important factor in relieving the pressure on the primary supply and a greater circularity leads to a lower criticality. It should be mentioned, however, that some authors suggest that the circular economy practices may not always lead to a lower criticality or more sustainability and an analysis and guidance is needed on a per-case basis [34]. 

## 4. Zero-Emission Vehicles (ZEV)

Governments, leading auto manufacturers and organizations with an influence over the future of the automotive industry and road transport committed to rapidly accelerating the transition to zero-emission transportation. A declaration of the 2021 United Nations Climate Change Conference COP26 pledges to work towards all sales of new cars and vans being zero emission globally by 2040, and by no later than 2035 in leading markets. United States seek to make half of new auto fleet electric by 2030. Some nations as Norway or Netherlands announced more aggressive timelines to phase out fossil fuel-powered cars by 2025–2030.

### 4.1. California’s Zero-Emission Vehicle Mandate

California has been the pioneer state and first established a ZEV mandate in 1990, issued by the California Air Resources Board (CARB). The California Zero-Emission Vehicle (ZEV) rule is seen as the most progressive and by some authors also as the most controversial air-quality policy ever adopted [35]. Adopted in 1990 as part of the Low-Emission Vehicle (LEV) program, and subsequently known as the ZEV mandate, it required major auto companies to ‘‘make available for sale” vehicles with zero criteria-pollutant tailpipe emissions.

The terminology for ZEV, which are primarily powered by an electric drive, varies by region. Electric vehicles are referred to as “new-energy vehicles” (NEV) in Chinese regulations, as “low-emission vehicles” (LEV) or “zero low-emission vehicles” (ZLEV) in the EU norms, and as “zero-emission vehicles” (ZEV) in U.S. regulations [36]. The ZEV regulation requires automakers to sell a minimum number of ZEV or risk paying a fine to maintain their sales operations in the state. The policy first took effect in 2005 and has since been implemented in nine other states in the United States. In Canada, Québec was the first and only jurisdiction outside the U.S. to have implemented its own version of the policy, which it introduced in 2016 [37].

### 4.2. Focus of Present Definitions 

A zero-emission vehicle is understood as any transportation vehicle using a propulsion technique, which does do not produce polluting exhaust. The essence of the present definitions of ZEV is that they consider only a portion of the use stage of the entire life cycle of the vehicle. No harmful pollutants (tailpipe emissions) during the use stage means that vehicles do not have exhaust system at all, such as battery electric vehicles (BEV), or they exhaust pollutant-free water vapor, such as hydrogen fuel-cell vehicles (FCEV). The majority of zero-emission vehicles employ a highly efficient electric drive system integrating the high-voltage storage batteries, a high-speed charging system, and one or more forms of onboard electric power generation, such as brake regeneration or solar panels. It should be mentioned that a vehicle charging is associated with some transfer losses from plug to battery, resulting in emitted heat from the device, assumed to be 10% for BEV [38]. Separate definitions of ZEV are specified by national and state/provincial institutions that offer procurement incentives to stimulate electric vehicle sales [39]. To allow the administrative execution of such widely leveraged policies, makes and configurations of vehicles covered are specified in detail.

Some vehicles generate residual emissions while engaging their gasoline-powered engines, such as hybrids (HEV) or plug-in hybrids (PHEV). As such, they are not true zero-emission vehicles. To avoid confusion, it should be noted that a term of partial zero-emission vehicle (PZEV) is also circulating, promoted by Subaru Corporation. The PZEV vehicles employ an internal combustion engine with claims of reduced smog-forming emissions through modifications in the areas of catalytic converters, fuel injectors, dual-filtration air-intake system and engine control module. 

### 4.3. Net-Zero Economy—Life Cycle Emissions

While internal combustion engine (ICE) vehicles generate energy during the internal combustion of hydrocarbons, EV batteries just store it (excluding a fraction generated during breaking). Therefore, energy has to be transferred from another source during charging. To achieve zero-emission during the vehicle use stage, it would require a charging network that is powered by a renewable energy grid. It means that the electricity is generated using the fossil-free resources, such as solar or wind power. Moreover, two other constantly overlooked components of the vehicle life cycle should be included into emission considerations, the manufacturing and end-of-life stages, as it is often termed “cradle-to-grave” approach. 

There is an environmental impact of electric car batteries during their production, use and disposal. Since most electric vehicles are powered by lithium-ion batteries, the key question is how much CO_2_ is emitted by manufacturing batteries. The production of a lithium-ion battery cell requires sourcing of as much as 20 different materials from around the world [40]. The mining and refining of battery materials and manufacturing of cells, modules and pack require significant amounts of energy, which generate GHG emissions. Mining lithium is water-intensive and contributes to air, soil and water pollution. For hard-rock mining, 15 tonnes of CO_2_ are emitted into the air for every tonne of mined lithium [41]. According to [40], the climate impact of a lithium-ion battery ranges from 39 kg CO_2e_/kWh to 196 kg CO_2e_/kWh. According to the review in [42], it ranges from 30 to 494 CO_2e_/kWh. For a Tesla Model 3 that holds an 80 kWh lithium-ion battery, the battery manufacturing emissions range between 3120 kg and 15,680 kg of CO_2_ [41]. 

It is commonly accepted that EVs generate considerably lower emissions over their lifetime than vehicles running on fossil fuels, regardless of the source of electricity used for their battery charging. For the electric grid of the United States, being a mixture of fossil fuels and low-carbon energy such as wind, solar, hydropower and nuclear power, driving an electric car releases less CO_2_ than driving a gas-powered car. However, for a power grid generated with coal, an electric vehicle has the fuel economy equivalent in the order of about 50 to 60 miles per gallon equivalent [41]. According to an assessment by the tool developed [43], an electric car with a battery produced in China where coal is the primary energy source and driven in Poland emits 37% less CO_2_ and a car with a battery produced in Sweden and driven in Sweden, emits 83% less CO_2_ than a car using a petrol engine. According to [44], that amount is 25–50% less than petrol cars estimated at around 24 tonnes of CO_2_ during their lifetime. 

To achieve the net zero-emission target from the entire life cycle of the vehicle, first, sustainable manufacturing is required with the creation of a circular supply chain by retrieving, recycling and reusing the battery raw materials to reduce the carbon emission to a minimum [45,46]. Then, the eventual remaining positive emission balance should be offset by activities leading to the equivalent amount of carbon removal.

## 5. Materials for Zero-Emission Vehicles

**Figure 7:**
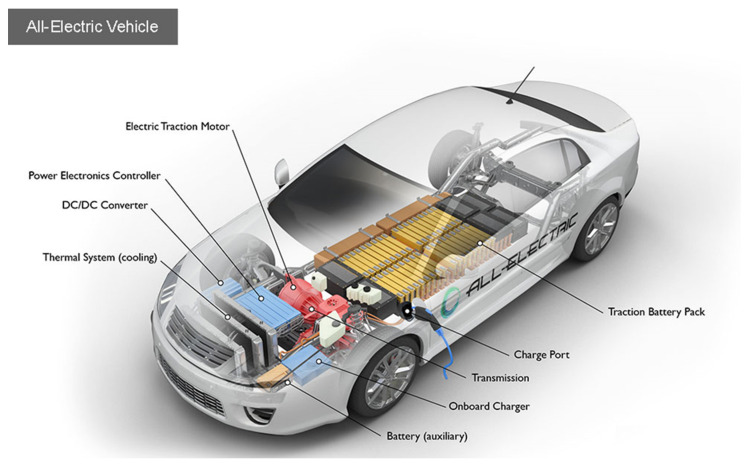
Key components of an all-electric car [48].

Although some materials used in electric drive vehicles are practically the same as those used in modern ICE vehicles, there are essential differences [47]. As shown in Figure 7, in an electric vehicle, the powertrain is completed through an electric motor, battery pack, inverter, on-board charger, DC–DC converter, and others, all requiring unique materials, with those used for major components of ZEV listed in Table 1.

As stated in the Introduction, according to the IEA, the typical electric car requires six times the mineral inputs of a conventional car [1]. It is clear that such high requirement of metals is not caused by just an increase in the vehicle weight. It is true that the overall weight of current BEV is higher than their ICE equivalents. The extra weight in an EV is largely due to the battery pack, shielding for the batteries, a heavier body, framework and stronger suspension. Currently, half of the EVs on the road weigh between 1500 kg (3300 lb) and 2000 kg (4400 lb), which is on average 340 kg (750 lb) more than an ICE vehicle of the same class [49]. This difference is not the reason for the six times larger quantity of mineral inputs required for electric cars assessed by the IEA.

### 5.1. Materials for Batteries

The battery is the essential energy storage system for BEV, PHEV and HEV that is also the single biggest cost component, reaching between one third and one quarter of the total vehicle production cost. The EV traction battery materials market is predicted to exceed $52 billion by 2031 [50]. For each battery type, the size controls its capacity. The smallest batteries today are around 30 kWh, whereas the largest range up to 100 kWh. The average weight of an electric car battery is 300 kg, but it may be twice as much on some models. The typical battery is composed mainly of metals in the active part of the cell but also of plastics, solvents and electronics. The battery technology is evolving rapidly and in the near future there will be a wider range of battery types, each demanding different minerals. A sharp increase in demand for battery materials is expected, e.g., for lithium and graphite by 9–10 times by 2030. Therefore, the key challenge is to reduce the amounts of minerals that need to be mined for EV batteries.

#### 5.1.1. Battery Cell Materials

At present, there are two types of electric car (traction) batteries commonly used:

- Lithium-ion battery (LIB), used by most EV vehicles;
- Nickel–metal hydride battery, used by hybrid vehicles.

**Figure 8:**
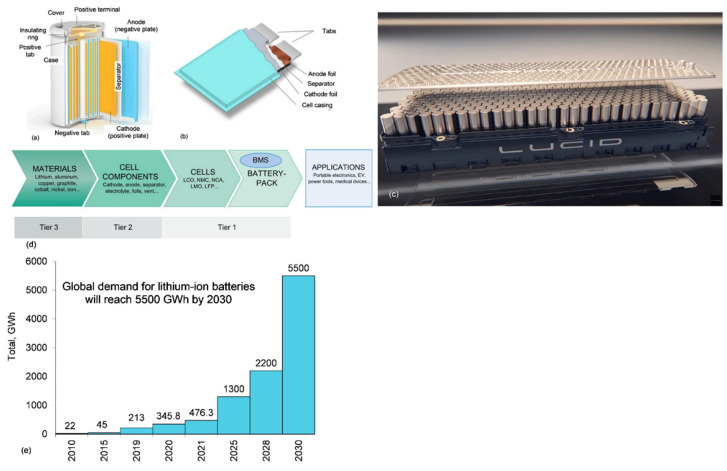
Lithium-ion battery for EVs: cell configuration with cylindrical anodes and cathodes (**a**) or flat ones sandwiched together (**b**), with an electrolyte filling the space between [53]; (**c**) Lucid Air battery module composed of cylindrical cells [52]; (**d**) production structure of the LIB industry, from raw materials to final applications [53]; (**e**) global demand for LIB with a 2025–2030 prediction (based on various sources).

As a technological component, lithium-ion batteries present a huge global potential towards energy sustainability and substantial reductions in carbon emissions. The first lithium battery was built in the 1970s with lithium metal and titanium sulphide as electrodes [51]. As seen in Figure 8a,b, the battery comprises essentially four components: cathode, anode, electrolyte and separator. Cylindrical cells are preferred by some manufacturers partly for their more consistent cooling behavior. Individual cells are arranged into modules (Figure 8c), e.g., a 644 km (400-mile) range would require 22 modules with 300 cells each, adding up to a total over 6000 cells [52]. 

The commercial batteries are named from the lithium-ion donator in the cathode, since it essentially determines the cell properties. Out of several lithium metal oxides used, the lithium nickel cobalt aluminum oxide (NCA) and lithium nickel manganese cobalt oxide (NMC) are the one used in EVs. For the Tesla Model S 75D with a weight of 2108 kg, an NCA battery with a capacity of 75 kWh weighs 506 kg. For a GM Chevrolet Bolt EV with a weight of 1624 kg, an NMC battery with a capacity of 60 kWh weighs 438 kg. 

The production structure of the Li-ion battery industry is shown in Figure 8d. It is predicted that the global cumulative lithium-ion battery capacity could rise over tenfold to 5500 gigawatt-hours (GWh) between 2021 and 2030 [54]. In 2021, the Asia Pacific region, led by China, accounted for 90% of the world’s battery manufacturing. It is expected that by the decade end this share will reduce to 69% while North America’s cell capacity could expand 10-fold. Europe is on track to overtake North America in 2022 and will account for over 20% of the global capacity by 2030. According to a newly released Li-ion battery database, global shipments in 2021 equated to 476.3 GWh, amounting to a 72.6% increase on 2020 [55]. The global demand for LIB with a 2025–2030 prediction is shown in Figure 8e.

**Figure 9:**
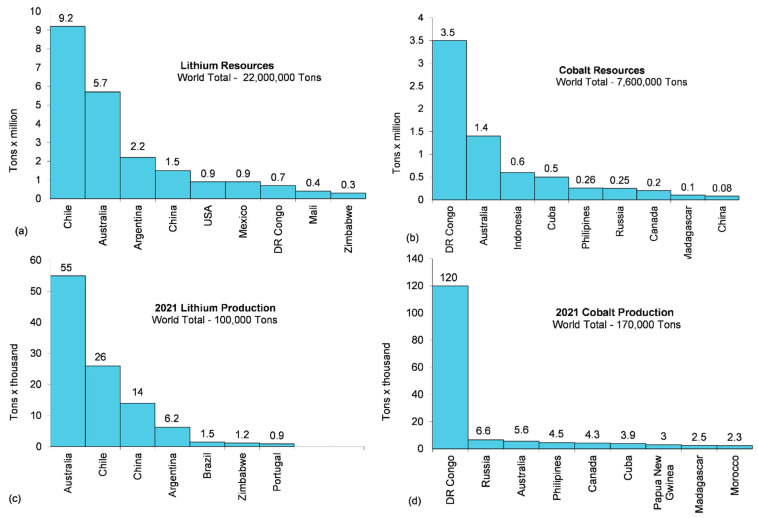
Main geographical locations of lithium (**a**) and cobalt (**b**) resources along with top 10 producers of lithium (**c**) and cobalt (**d**) in 2021, emphasizing the lower resources of cobalt accompanied by its larger production. Based on data from [26].

Lithium, cobalt, nickel and graphite are integral materials in the composition of lithium-ion batteries for electric vehicles. Table 2 lists the characteristics of these elements and indicates their natural abundance rank, current global reserves and annual production as well as the demand share of the Li-ion battery sector [53]. Two elements stand out from this list with near-critical and critical status: lithium and cobalt. Their main resource geographical locations and top 10 producers in 2021 are shown in Figure 9a–d. As cobalt is the most critical element here, there is a continued shift away from the dominant cobalt-rich LIB types and a broader shift away from LIB technology with lithium–sulfur (Li-S) pointed out as the most prospective candidate.

#### 5.1.2. Battery Pack Materials

In addition to the battery improvement at the cell level, opportunities exist to an increase in energy density through design control at the pack level [56]. Two directions combining the design and material are pursued. The first one is improving the thermal management of the battery pack that allows for a higher energy density and fast charging without a danger of fire. This is achieved through composite enclosures, fire-retardant materials and thermal interface materials. The second one is lightweighting, achieved through a reduction of the weight of materials used around the cells, allowing for a lighter battery pack or more cells used for the same weight.

Forecasted battery pack materials include aluminum, copper, thermal management materials, thermal interface materials, steel, glass-fiber-reinforced polymers, carbon-fiber-reinforced polymers, intercell insulation, compression foams and housings and packed fire-retardant materials [50].

### 5.2. Materials for Electric Traction Motors

**Figure 10:**
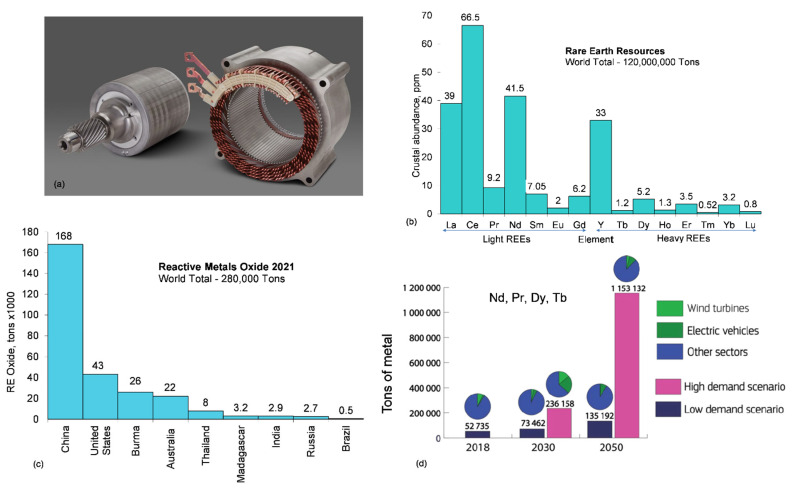
Materials for electric traction motor: (**a**) GM EV motor with Ultium rotor equipped with permanent magnet [57]; (**b**) rare earth elements found in natural deposits—the “lanthanides” plus yttrium, data from [58]; (**c**) world mine production of rare earth elements in 2021, expressed in metric tons of oxides, data from [26]; (**d**) estimated global demand for neodymium, praseodymium, dysprosium and terbium for wind turbine generators, motors for electric vehicles and other sectors, according to low- and high-demand scenarios [59].

The majority of EV models use synchronous permanent magnet motors, which became widespread industrially in the 1990s and are smaller and more efficient than induction asynchronous motors, the common alternative (Figure 10a). Their advantages of a very high efficiency, lower cooling needs and high power density (both gravimetrically, kW/kg and volumetrically, kW/cm^3^) allows another fundamental component, the battery, to be smaller, thus reducing costs. There are four compositions of rare earth permanent magnets, SmCo_5_, Sm_2_Co_17_, SmFeN and NdFeB, with the latter one being the most widely used in EV traction motors. The requirement for rare earths in motor magnets, create a number of mineral supply challenges.

#### 5.2.1. Nd-Fe-B Magnets with Heavy Rare Earths

The essential motor component, the neodymium magnet, also known as NdFeB, NIB or Neo magnet, developed in 1982, is produced by sintering metallurgical powders with a ration adjusted to the chemical composition of the alloy required and either cut from isostatically pressed blocks or die-pressed. Their excellent magnetic properties can be traced back to the strongly magnetic matrix phase Nd_2_Fe_14_B exhibiting a high saturation polarization and a high magnetic anisotropy. Typically, the alloys contain between 60–70 wt.% iron, 28–35 wt.% REEs (Pr, Nd, Tb, Dy), 1–2 wt.% boron and 0–4 wt.% cobalt [60].

Magnets destined for EV traction motors contain 8–10 wt.% of the heavy rare earth element dysprosium with the purpose to improve the magnet’s resistance to demagnetization and increase its service temperature. For traction motor applications, the resistance to demagnetization is a critical performance as these magnets operate in high-temperature environments with strong demagnetizing fields. Thus, the amount of Dy is influenced by the demagnetization stress and operating temperature. For 11 wt.% Dy, magnets can be used up to 220 °C. Although terbium is more effective than dysprosium in this respect, it is also less abundant, leaving the former one as the choice alloying option. Praseodymium can directly substitute neodymium to some extent without severe impact on the magnetic properties. Cobalt is commonly added to improve the corrosion resistance.

#### 5.2.2. Heavy-Rare-Earth-Free Nd-Fe-B Magnets

While Nd is a relatively abundant light rare earth element, the annual production of the heavy rare earths Dy and Tb is only 1.4% and 0.1% of that of Nd, respectively, [61] (Figure 10b). Due to the scarcity and high demand, Dy and Tb have been facing a high price volatility, which led to a strong effort by manufacturers to design magnet compositions without Dy and Tb or at least with their contents reduced. As a result of research, two ways to improve coercivity with reduced or no HREEs were identified: (1) utilizing the grain boundary diffusion (GBD) process, and (2) controlling the microstructure [62]. The GBD process has been applied to sintered Nd-Fe-B magnets to effectively reduce HREE usage by more than 50%. Controlling the microstructure in terms of reducing grain size and promoting grain isolation magnetically have been generally known to increase coercivity. Thus, hot-deformed Nd-Fe-B magnets are considered promising candidates to achieve completely HREE-free Nd-Fe-B magnets with high performance. The downside is that the coercivity of the hot-deformed magnet is not as high as expected given its fine grain size. 

Toyota adopted new technologies that suppress the deterioration of coercivity and heat resistance, even when neodymium is replaced with lanthanum and cerium, and developed a magnet that has equivalent levels of heat resistance as earlier neodymium magnets, while reducing the amount of neodymium used by up to 50% [63]. As an alternative, the flash spark plasma sintering (FSPS) process has been applied to a Dy-free Nd30.0Fe61.8Co5.8Ga0.6Al0.1B0.9 melt spun powder [64,65].

#### 5.2.3. Heavy REE Supply and Demand 

Rare earths are relatively abundant in the Earth’s crust, but minable concentrations are less common than for most other ores (Figure 10c). The REEs are divided into two groups on the basis of their atomic weight: (a) the light REEs (LREEs), which include lanthanum through gadolinium (atomic numbers 57 through 64) and (b) the heavy REEs (HREEs), which include terbium through lutetium (atomic numbers 65 through 71). Although yttrium is considered a light element based on its atomic number of 39, it is traditionally placed with the HREE group because of its common chemical and physical affiliations. In North America, measured and indicated resources of rare earths were estimated to include 2.7 million tons in the United States and more than 15 million tons in Canada [26].

It is predicted EVs will consume one-fourth of the global NdFeB magnets production by 2030 [59] (Figure 10d). According to a global critical minerals and metals research firm [66], the demand for rare earth magnets will outpace the supply of neodymium, praseodymium and dysprosium oxides. The market for magnet’s rare earth oxides is set to increase three times by 2035 with a total demand forecasted to increase at a CAGR of 8.3%. As a result of an undersupply of neodymium, praseodymium, dysprosium and terbium oxide, an annual NdFeB shortage of 206,000 tonnes (one third of the total market) is anticipated. Due to a lack of new primary and secondary supply sources, global shortages of neodymium, praseodymium and didymium oxide will reach 68,000 tonnes by 2035. 

### 5.3. Materials for Car Body 

**Figure 11:**
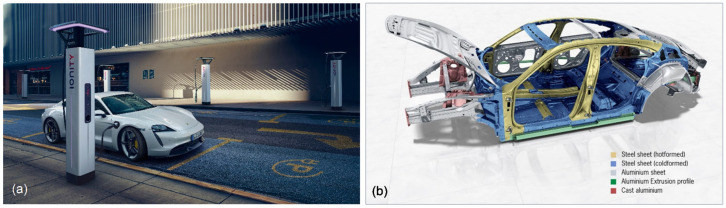
The electric car (**a**) and its body as a combination of steel and aluminum alloys obtained by different processing routes, well-designed for maximum strength (**b**), Porsche Taycan [68].

As electric vehicles have an electric motor and a battery instead of a combustion engine and a fuel tank, they have fundamentally different architecture. The need to secure a large, heavy battery pack at the bottom of the vehicle and the desire to use one platform for multiple vehicles will drive the automotive industry back into a body-on-frame arrangement. It is of interest that the major materials used for an EV’s body are similar to those used for modern ICE vehicles. As described recently [67], automotive lightweighting is becoming the mature growth trend, driven by sustainability, cost and performance, that creates the enormous demand for modern lightweight materials. It appears that the emergence of electric vehicles creates even more pressure on lightweighting. A quest for lightweight materials creates many challenges and opportunities not only for existing conventional but for novel metallic alloys. Due to diverse compositions of many modern alloys, alloy information helps prioritize material criticality lists. The optimal designs promote the multimaterial approach, where the best characteristics of different materials are utilized [68] (Figure 11).

#### 5.3.1. Ferrous Alloys 

At present, ferrous metals, mainly steel, maintain the majority share of the automotive market, with about 70% of an average car weight consisting of steel sheet metal, forged steel parts and cast iron. The steel offers the attractive features of low cost, manufacturability, recyclability and the availability of specialized alloys [69]. 

During the last five decades, three generations of Advanced High-Strength Steel (AHSS) have been developed for the purpose of lightweighting in the automotive industry. The AHSS grades are stronger than the conventional automotive steel, which allows a thinner sheet than the conventional one, which enables automakers to build vehicles that are both strong and lightweight. It is anticipated that they will dominate the vehicles’ material share in the future, especially in body-in-white applications, offering a weight reduction of the vehicle body by as much as 25%. According to the Steel Market Development Institute, with an addition of the third generation of AHSS, that weight reduction could reach 30–40%. Although iron is not listed as a critical mineral, many alloying additions of steel such as V, Cr or Mn are included in that list (Table 1).

#### 5.3.2. Aluminum Alloys

Aluminum alloys offer a lower-weight alternative to steel, and they fit greatly into a circular economy due to their high recovery and reusability in new products. The current North American Light Vehicle Aluminum Content and Outlook report reveals that aluminum is the fastest growing automotive material. The aluminum usage in automotive has grown from 154 kg per vehicle in 2010 to 208 kg per vehicle in 2020 [70]. A further growth is anticipated to 233 kg per vehicle by 2026, up by 12% from 2020 levels. According to European data [71], the amount of aluminum increased from 50 kg per vehicle in 1990 to 151 kg at present with a projection of reaching 196 kg per vehicle by 2025. By 2030, the aluminum demand from electric vehicles will near 10 million tonnes, a tenfold increase from 2017. Extrusions and rolled aluminum products will contain much higher contribution of primary metal than it is in internal combustion engine vehicles today. Practically all casting alloys use Sr as a modifying agent. Many aluminum alloys contain transition metals or rare earth metals as alloying elements [72] and are included into the critical minerals list (Table 1).

#### 5.3.3. Magnesium Alloys

Magnesium alloys are 75% lighter than steel, 50% lighter than titanium and 33% lighter than aluminum and due to their lightweighting characteristics have proven to be attractive structural materials for transport vehicles. In contrast to extensive research projects and promising industrial applications at the beginning of the previous century by Volkswagen and Porsche, the anticipated usage of 50 kg per car in 2015 did not materialize. At present, the automotive use of magnesium is still well beyond the high-volume commercial applications [73]. Due to excellent castability of magnesium alloys, which exceeds other metals such as aluminum and copper, casting is the dominant manufacturing process for magnesium components, representing about 98% of all structural applications for magnesium [74]. Development of low-cost magnesium sheet is seen as an enabler to downstream processing required to expand the existing magnesium applications [75].

#### 5.3.4. Substitution Dilemma of Critical Materials and Lightweighting Goals

To reduce the consumption volume of critical minerals they should be substituted with other elements outside the critical list. According to this strategy, aluminum and magnesium alloys should be substituted with ferrous alloys, classified as noncritical materials. Such a substitution, however, would lead to an increase in the vehicle weight and reduce its driving range. A compromise is, therefore, required to satisfy both strategies with lightweighting goals being generally seen as the top priority.

### 5.4. Auxiliaries

Electric vehicles have a number of auxiliary systems. Although some systems operate the same way as they do in ICE vehicles, other systems, such as the power steering and power brakes, require an additional small electric motor and have a minor impact on the vehicle range. The same is true with lights and defroster, which together with the former affect the driving range up to 5%. However, the air conditioning and heating systems on electric vehicles are different and can have a dramatic impact on the range by up to 35%. Heat pumps similar to those used in homes are being used on the latest electric vehicles; these reduce the power requirements for heating and cooling. An example is the General Motors EV1, which uses an electrically driven heat pump for climate control. In contrast to ICE, electric vehicles have a propulsion motor that is not constantly running, means the auxiliaries must be independently powered [76]. 

#### 5.4.1. Twelve-Volt Lead Auxiliary Batteries

Other important components of electric vehicles are 12 V lead-acid batteries, invented in 1859, that perform the secondary role of powering the auxiliary systems managing the human interface. They also provide back-up power for safety relevant features such as power steering and brake boosting and for the vehicle’s comfort features, such as radio and sound systems. Although Tesla announced it will replace the auxiliary lead batteries with 12 V (possibly also 48 V) lithium-ion, having 2–3 times longer lifespan, in future versions of its models S, it is anticipated that lead batteries will remain critical to the vast majority of electric vehicles. 

The global lead market is mature with roughly 12 million tonnes of lead being produced and consumed every year with 85% used by batteries. This equates to around 150 times the volume of the nascent lithium market, and 80 times the global cobalt market [77]. Due to very high battery recycling rate (over 95%) of the 12 million tonnes of lead, only 4.5 million tonnes come from primary production, with the rest coming from recycling. Although lead is not listed as critical mineral, positive grids of lead-acid batteries are made of lead-5-11 wt.% antimony alloy and the latter element is present on lists of all 5 countries considered in Table 1.

#### 5.4.2. Copper Conductors in EV

**Figure 12:**
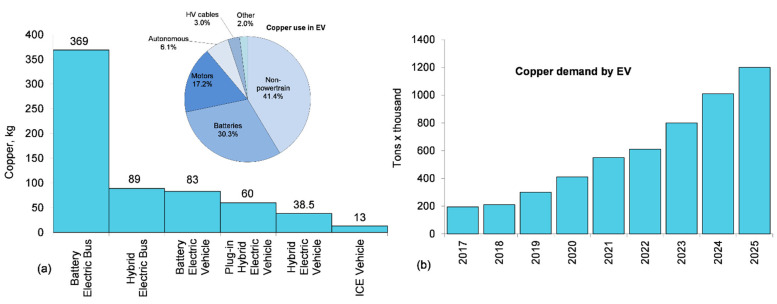
Copper use in EVs: (**a**) per vehicle content in ICE and EVs with an example of internal distribution for EVs; (**b**) growth trend of Cu use for EV industry, based on data from [78,79].

Copper is essential to electric vehicle technology and is used in electric motors, batteries, inverters, wiring, charging stations and supporting infrastructure. A typical EV contains 10 times more copper than an ICE vehicle. It is anticipated that the EV growth will have a substantial impact on copper demand increase by 1700 kilotons by 2027 [78], where supply will struggle to match upcoming demand (Figure 12). 

Copper applications for an EV expand beyond the vehicle to the charging infrastructure. According to SAI Industrial, the number of EV charging ports is predicted to grow from 3.2 million in 2021 to 152.3 million in 2040. This growth is expected to increase copper demand in the sector to reach 978,000 t in 2040—a dramatic increase from the 43,300 t used in 2021 [79].

#### 5.4.3. Aluminum Conductors as an Alternative to Copper 

In the last two decades all-aluminum alloy electrical grade conductors (AAAC) have been increasingly used as an alternative to copper. To reduce the weight of the wiring harness, aluminum has been increasingly considered instead of copper for its low specific gravity. The use of aluminum wires is also effective for cost reductions. The conductor strength of conventional aluminum wires is insufficient, it is impossible to use them in small sizes such as 0.35 mm^2^ or 0.5 mm^2^, which are the sizes of conventional copper wires, in engine compartments that are subjected to strong vibration, so an increase in strength is required [80,81]. In general, an increase in strength of Al wires results in a reduction of electrical conductivity as is the case of Al–Mg–Si, Al–Fe–Si, Al–Fe–Cu alloy wires, which lose over 50% electricity as compared to the International Annealed Copper Standard (IACS), creating electricity waste during the transport process [82]. In this respect, transition or rare earth alloying additions are being researched, in particular scandium and cerium [83,84]. The Al–0.12Sc–0.04Zr alloy after heat treatment reached an ultimate tensile strength of 160 MPa and an electrical conductivity of 64.0% (IACS) [85]. As another option, Al-Zr alloys have prospects in electrical engineering applications but the low nucleation intensity of the Al_3_Zr particles is a drawback of these chemistries [86]. The good combination of strength, plasticity and electrical conductivity of the investigated AlYSc02 alloy makes it a promising material for electrical conductors with a yield stress of 180 MPa, tensile strength of 200 MPa, elongation of 15% and an electrical conductivity of 60.8–61.5% (IACS) [87].

## 6. Recovering the Critical Minerals from EV

A transition from hydrocarbons in ICE vehicles to minerals in ZEV brings a fundamental change to the recycling strategy: in contrast to hydrocarbons which are consumed during combustion, the minerals can be recycled and reused. Recycling the components common in ICE and EVs has been implemented for decades. The challenges are with recovering minerals from components unique to EVs.

### 6.1. Recovering Metals from Lithium-Ion Batteries

A circular economy with an environmentally sound recycling approach is needed for electric vehicles since batteries pose challenges in terms of fires and hazardous contamination [88]. It has been estimated that under idealized conditions, recycling end-of-life electric vehicle batteries could provide 60% of cobalt, 53% of lithium, 57% of manganese and 53% of nickel needed globally in 2040 [89]. At present, however, recycling technologies are still under development and an insufficient volume of LIB batteries for recycling is available to make the process profitable [90,91].

**Figure 13:**
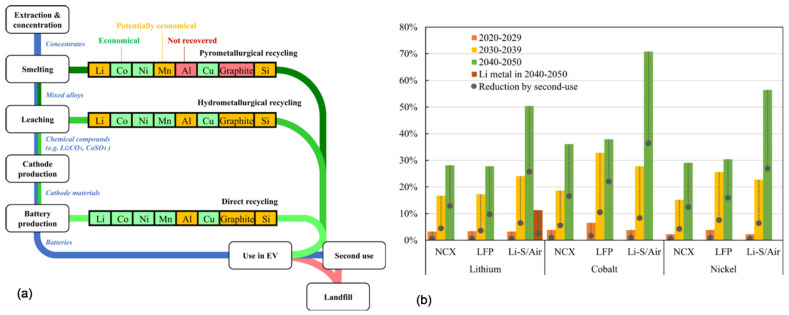
Schematic showing how the three recycling scenarios of battery recycling, work together in a closed loop and which materials are recovered (**a**) and closed-loop recycling potential of battery materials for next three decades (**b**); gray dots show how second use, which postpones the time of recycling, reduces the closed-loop recycling potentials and the availability of secondary materials in the coming decades [92].

Current commercial recycling technologies for LIB batteries include pyrometallurgical method that involves smelting entire batteries or, after pretreatment, battery components and hydrometallurgical processing that involves acid leaching and subsequent recovery of battery materials, e.g., through solvent extraction and precipitation [92]. As a third option, a direct physical recycling is listed. Although the pyrometallurgy is a simple process, it allows the recovery of cobalt and nickel when aluminum, manganese and lithium are consumed and end up in a slag. The summary of the method consisting of three potential recycling scenarios, pyrometallurgical, hydrometallurgical and direct recycling for NCX and LFP batteries, as well as mechanical recycling for Li–S and Li–Air batteries and closed-loop recycling potential of battery materials for the 2020–2029, 2030–2039, and 2040–2050 periods are shown in Figure 13.

In closed-loop recycling, pyrometallurgical processing is followed by hydrometallurgical processing to convert the alloy into metal salts. Although still in early development stages, a direct recycling that aims at recovering the cathode materials while maintaining their chemistry could be economically and environmentally advantageous. In a direct recovery, components are separated by a variety of physical and chemical processes, and all active materials and metals can be recovered.

### 6.2. Recovering Graphite from Lithium-Ion Batteries

Of the most common carbon materials in the LIB anodes, such as graphite, soft carbon, hard carbon and carbon nanotubes, graphite dominates because of its special layered structure that facilitates the relatively stable insertion and extraction of Li^+^ ions. Due to the high consumption of graphite in lithium-ion batteries its recycling could compensate for the shortage of graphite resources in production. At present, two methods for recycling the spent graphite in the pretreatment stage are listed: direct crushing and artificial splitting [93]. A method to regenerate spent graphite via a combined sulfuric acid curing, leaching and calcination at 1500 °C was also proposed [94]. In another recovery idea, the electrolyte and binder in spent LIB materials were completely removed by roasting under an air atmosphere at 505 °C, which significantly improved the floatability difference between graphite and cathode materials [95]. As a result, graphite and cathode materials were separated by flotation. However, the present methods for recovering graphite are not feasible for large-scale industrial processes due to excessively high costs caused by economic and environmental constraints.

### 6.3. Recycling the NdFeB Magnets from Electric Drive Motors

Permanent magnet synchronous motors contain considerable amounts of rare earth elements that cannot be recovered in conventional recycling routes and their recycling could have large economic and environmental benefits. In general, recycling processes for NdFeB magnets can be classified into direct reuse, reprocessing of the alloys, and raw material recovery [96]. The flow sheet of the hydrometallurgical recycling processes was developed in the MORE project in Germany with costs and revenues for the magnet extraction and processing to neodymium and dysprosium oxides [96,97]. The project showed that electric drive motors including the NdFeB magnets can be recycled but many challenges and uncertainties remain.

The potential for Nd and Dy recovery from EVs and other equipment containing these elements, such as computer hard drives (HDD) and magnetic resonance imaging (MRI), in the US was determined in [98]. The factors used in the analysis were the number of units manufactured each year, the NdFeB magnet mass in each unit and the REE composition of the magnets employed. The conclusion reached was that the recovery of rare earths from EoL products in the U.S. could only partially address the risks in the rare earths supply. This is caused by China’s domination of most of the downstream processing steps in rare earth permanent magnets manufacturing. 

## 7. Concluding Remarks

An anticipated implementation of large quantities of minerals, in particular metals, into the manufacturing of strategic components of zero-emission vehicles will likely bring new challenges to energy security. As a result, a reduced dependency on conventional hydrocarbon resources, may lead to new and unexpected interdependencies, including dependencies on raw materials. It is concluded that to minimize the impact of the metal-intensive transition to clean transportation, in addition to overcoming challenges in minerals exploration and processing, further progress in understanding the properties of critical materials will be required to better correlate them with the intended applications, to identify potential substitutions and to optimize their use through sustainable exploration of their resources and a circular economy.

## Figures and Tables

**Table 1:** Critical minerals lists, issued by selected countries along with minerals used in key components of zero-emission vehicles.

		Canada	USA	EU	China	Japan	Australia	Electric Vehicles		
		2021	2022	2020	2016–2020	2018	2022	Battery	Fuel Cells	Motor
1	Aluminum/bauxite	×	×	×	×		×			×
2	Antimony, Sb	×	×	×	×	×	×			
3	Arsenic, As		×							
4	Barite, Barium		×	×		×				
5	Beryllium, Be		×	×		×	×			
6	Bismuth, Bi	×	×	×		×	×			
7	Borate, Boron			×		×			×	×
8	Cesium, Cs	×	×			×				
9	Chromium, Cr	×	×		×	×	×		×	×
10	Coal, Carbon, C			×	×	×				
11	Cobalt, Co	×	×	×	×	×	×	×	×	
12	Copper, Cu	×			×			×	×	×
13	Fluorspar, fluorine	×	×	×	×	×				
14	Gallium, Ga	×	×	×		×	×			
15	Gas-natural				×					
16	Gas-shell				×					
17	Germanium, Ge	×	×	×		×	×			
18	Gold, Au				×					
19	Graphite	×	×	×	×		×	×	×	
20	Hafnium, Hf		×	×		×	×			
21	Helium, He	×					×			
22	Indium, In	×	×	×		×	×			
23	Iron, Fe				×					
24	Lithium, Li	×	×	×	×	×	×	×	×	
25	Magnesium, Mg	×	×	×		×	×		×	
26	Manganese, Mn	×	×			×	×	×	×	
27	Methane-coalbed				×					
28	Molybdenum, Mo	×			×	×				×
29	Nickel, Ni	×	×		×	×		×	×	
30	Niobium, Nb	×	×	×		×	×			
31	Oil				×					
32	PGM	×	× (1)	×		×	×		×	
33	Phosphate			×						
34	Phosphorus, P			×	×					
35	Potash	×			×					
36	REE-all	×	× (1)	× (2)	×	×	×		×	×
37	Rhenium, Re					×	×			
38	Rubber nat.			×						
39	Rubidium, Rb		×			×				
40	Scandium, Sc	×	×	×			×			
41	Selenium, Se					×				
42	Silicon metal, Si			×		×	×	×	×	×
43	Silver, Ag								×	
44	Strontium, Sr			×		×			×	
45	Tantalum, Ta	×	×	×		×	×			
46	Tellurium, Te	×	×			×			×	
47	Thallium, Tl					×				
48	Tin, Sn	×	×		×					
49	Titanium, Ti	×	×	×		×	×	×	×	
50	Tungsten, W	×	×	×	×	×	×			
51	Uranium, U	×			×					
52	Vanadium, V	×	×	×		×	×		×	
53	Zinc, Zn	×	×							
54	Zirconium, Zr		×		×	×	×		×	
	Total	31	50	30	24	34	26			

PGM—platinum group metals (6 elements): iridium, osmium, palladium, platinum, rhodium and ruthenium; REE—rare earth elements (17 elements): lanthanum (La), cerium (Ce), praseodymium (Pr), neodymium (Nd), promethium (Pm), samarium (Sm), europium (Eu), gadolinium (Gd), terbium (Tb), dysprosium (Dy), holmium (Ho), erbium (Er), thulium (Tm), ytterbium (Yb), lutetium (Lu), scandium (Sc) and yttrium (Y). (1) Listed individually as above, (2) Split into light REE and heavy REE.

**Table 2:** Essential elements for the Li-ion battery sector [26,53].

Element	Battery Component	Abundance Rank	Global Reserves	2020 Mine Production	Li Battery Industry Share	Critical Mineral List, Canada	Current Status [53]	Future Perspective [53]
			Mt	t/year				
Graphite	Conventional anode, electrolyte	15	320	966,000	Around 3%	Critical	Not critical	Not critical
Nickel	NCA, NMC cathode ^(1)^	24	95	2,510,000	1–2%	Critical	Not critical	Not critical
Cobalt	NCA, NMC cathode	32	7.6	142,000	30%	Critical	Critical	Critical
Lithium	All cathodes, electrolyte	33	22	82,500	39%	Critical	Not critical	Near-critical

(1) Cathode active materials: NCA—lithium nickel cobalt aluminum oxide (LiNiCoAlO_2_); NMC (NCM)—lithium nickel cobalt manganese oxide (LiNiCoMnO_2_).
